# Cultural differences in the measurement of self-curiosity within Mexico: a person-centered and variable-centered study

**DOI:** 10.12688/f1000research.140151.1

**Published:** 2023-09-18

**Authors:** Filippo Aschieri, Angélica Quiroga Garza, Giulia Pascarella, Michela Zambelli, Semira Tagliabue

**Affiliations:** 1 Universita Cattolica del Sacro Cuore, Milan, Lombardy, Italy; 2 Universidad de Monterrey, San Pedro Garza García, Nuevo Leon, Mexico; 3 Consultorio Interprovinciale di Assistenza Familiare, Fondazione Poliambulanza Istituto Ospedaliero, Brescia, Lombardy, 20100, Italy; 4 Universita degli Studi di Genova, Genoa, Liguria, Italy; 5 Universita Cattolica del Sacro Cuore, Brescia, Lombardy, Italy

**Keywords:** Self-Curiosity Attitude-Interest Scale, Latent Profile Analysis, Measurement invariance, Cultural profiles

## Abstract

**Background:** This study assessed the role of culture in the measurement of the Self-Curiosity Attitude-Interest scale (SCAI-M), a measure of attitude and interest in increasing one’s knowledge of self, adopting both a person-centered and variable-centered approach.

**Methods:** The study was conducted on a Mexican sample composed of 484 adult participants who completed both the SCAI-M and a series of instruments that measure cultural dimensions through Qualtrix. Data were collected between November 9, to December 18, 2020, and respondents were contacted using advertisements on social media platforms (Facebook and WhatsApp). Analyses included multigroup confirmatory factor analysis and latent profile analysis.

**Results:** A latent profile analysis allowed for the generation of three groups featuring distinct cultural orientations that were similar to previously found cultural profiles (Consensus-oriented Egalitarians, Flexible Individualists, and Rules-based Competitors). Multigroup Confirmative Factor Analysis showed partial metric and scalar invariance for the SCAI-M between groups; moreover, we found proofs of convergent validity with other cultural dimensions besides the ones linked with the Hofstede model. Our results indicate that cultural profiles and cultural variables are associated with both the level and meaning of self-curiosity among Mexican citizens.

**Conclusions:** Finally, the discussion includes considerations on self-curiosity divergence among minority cultures and relevant clinical applications; a field for which we propose future research.

## Introduction

In clinical settings, curiosity about oneself is a central construct for psychological assessment, psychopathology and intervention. In the field of psychological assessment, Finn (1996) proposed to engage clients in defining their assessment questions from the onset of the consultation, stressing that promoting clients’ curiosity about themselves and about the origins of their problems increases clients’ participation and utility of the assessment. Kashdan and Fincham (2004) linked curiosity with self-regulation, Borawski (2022) highlighted that loneliness and search for meaning in life are mediated by the capacity to reflect (i.e., develop curiosity about oneself). Finally, the capacity to reflect upon inner conscious thoughts and mental processes has been found as a fundamental component for the patients’ engagement in psychotherapy ( Conte *et al.,* 1990) and for their effectiveness ( Nyklíček *et al.,* 2020).

To formally assess how people observe and explore their inner world, the authors proposed the Self-Curiosity Attitude-Interest scale (SCAI). Its structure consists of two positively correlated factors, Attitude toward Self-Curiosity (ASC, disposition to explore their inner world, four items) and Interest in Increasing Knowledge of Self (IKS, interest in understanding better themselves, three items) ( *r* ranging from .35 to .50). The scale had acceptable reliability coefficients considering its short length ( *Cronbach alpha* across studies ranging from .58 to .69 for ASC and from .63 to .72 for IKS) and showed convergent validity coefficients with reflection ( *r*=.60), openness, awareness, and motivation toward knowledge ( *r*=.40), and general curiosity, interest, and desire for stimulating experiences ( *r*=.30). Test-retest reliability for IKS was lower ( *r*=.67) than for ASC ( *r*=.81), indicating that the former is more sensitive to changes, whereas the latter behaves more like a stable trait-like feature. Discriminant validity analyses showed self-curiosity to be unrelated both to fluid intelligence ( Aschieri *et al.,* 2016) and current emotional states ( Aschieri and Durosini, 2015).

Self-curiosity could be linked with people’s cultural characteristics. The construct of curiosity itself can be considered a cultural trait ( Lindholm, 2018), and its development varied across different cultures: western cultures welcome its early presence, while non-western cultures value more obedience and respect. Among adults, an international survey found curiosity to be one of the most important character strengths; however, its ranking varied among cultures. Indeed, in some cultures curiosity was the most important character strength (e.g., Switzerland, Japan, Denmark), in other cultures it was in the low half of the top ten most important strengths (Brazil, Saudi Arabia, Sri Lanka), and in some other cultures curiosity had an intermediate value (Paraguay, Pakistan, and Turkey; McGrath, 2015).

Due to the expected role of culture on self-curiosity, the SCAI has been studied in different countries. The SCAI’s two-factor structure was replicated in Czech Republic, Colombia, Mexico, and Japan. However, the scale incorporated modifications due to the respondents’ culture. For example, in the SCAI-J ( Ushiyama *et al.*, 2022) all negative items related to the IKS were substituted with new items, all positively keyed. The application of the SCAI to non-Italian respondents largely confirmed the same pattern of concurrent validity found in Italy. The SCAI Scale correlated with reflection ( *r _Japan_* =.43); self-awareness ( *r _Colombia_* =.38, *r _Mexico_* =.20), interest and deprivation factors of general curiosity ( *r _Colombia_* equal to, respectively, .30 and .23; *r _Mexico_* equal to, respectively, .26 and .12), personality traits (Openness, *r _Colombia_* =.34; *r _Mexico_* =.24; *r _Japan_* =.16), and learning motivation (intrinsic motivation Towards Knowledge, *r _Colombia_* =.35; Intrinsic Motivation Towards Stimulating Experiences, *r _Colombia_* =.30; Intrinsic Motivation Towards Achievement, *r _Colombia_* =.24). The administration of the SCAI in different countries showed also differences in the strength of its relations with other variables, such as Extraversion ( *r _Colombia_* =.20, *r _Mexico_* =-.04; *r _Japan_* =-.02) and Agreeableness ( *r _Colombia_* =.15,** *r _Mexico_* =.14; *r _Japan_* =.04).

Previous studies found full metric invariance, but a lack of scalar invariance ( Durosini *et al.*, 2018; Aschieri *et al.*, 2021). The lack of scalar invariance affects the final score, so it seemed that people belonging to different countries could obtain different levels of self-curiosity due to differences in the measurement of the construct.

These findings indicated that self-curiosity possessed some differences in the measurement process linked with cultural aspects. Importantly, all the aforementioned studies were conducted with a unit of analysis based on the country, assuming that the country was representative of a specific culture. However, it has been recently underlined that the country is not always a good proxy for cultural differences in values, and that many of those differences are within-country and more linked with other demographical or environmental factors ( Taras *et al.,* 2016). Often, studies that highlight the importance to investigate within-country differences, adopt a person-centered approach by which different cultural profiles within one country can be found.

Recently, the latent profile analysis (LPA) technique has been applied to identify groups of individuals with similar cultural patterns of scores on dimensions of interest. This approach uses multiple attributes from each respondent to identify the boundaries that separate one group of individuals with similar cultural attributes from another, without defining the boundaries ex-ante. For instance, Cooper *et al.* (2020) investigated cultural profiles through LPA in 1071 participants from five different states (China, Germany, India, Russia, and the USA) using the Cultural Values Scale (CVSCALE, Yoo *et al.,* 2011) that measure the five dimensions of the Hofstede (2001)’s model of cultural differences: power distance, uncertainty avoidance, individualism/collectivism, masculinity/femininity, and long/short term orientation. Three different profiles were found. The first profile (‘flexible individualists’, 14% of the pooled sample) was characterized by moderate levels of masculinity and relatively low power distance, uncertainty avoidance and collectivism. They were very flexible and individualistic. The second profile (‘consensus-oriented egalitarians’, 46% of the pooled sample) was characterized by a significantly higher uncertainty avoidance and collectivism, and low power distance and masculinity. Members of this second group placed great importance on group consensus and typical values of egalitarian perspectives. The third profile (‘rules-based competitors’, 40% of the pooled sample) was characterized by high levels of power distance, masculinity, uncertainty avoidance, and collectivism. They placed an important emphasis on competition for power and status and preferred clear rules relating to hierarchies and social obligations. Another study, conducted on Latino immigrants in the United States ( Rojas *et al.,* 2021) also found three cultural profiles: low socio-cultural protection (30%), high socio-cultural protection (4%), and high socio-, low-cultural protection (66%). People in the first profile were characterized by the highest levels of machismo (a socially constructed set of behaviors guiding male gender role, De La Cancela, 1986, in Estrada *et al.,* 2011) along with the lowest levels of familismo (a cultural value that encompasses using family members as role models, turning to family members as sources of support, and prioritizing the family’s well-being over the individual’s ( Stein *et al.,* 2014 in Montoro and Ceballo, 2021), social-support, and multi-group identity, whereas people with the lowest levels of machismo and caballerismo (a code of masculine chivalry for a male adopting proper, respectful manners, Arciniega *et al.*, 2008) were the ones with the high socio-cultural protection. The high socio-, low-cultural protection profile showed the highest levels of familismo *.* Overall, these findings indicate that the cultural dimensions described in Hofstede’s model (2001) can effectively be used to construct groups of respondents with similar cultural profiles.

The possibility to identify groups based on respondents’ cultural orientation within the same country allows involving participants from a single nation to explore the relationship between psychological constructs such as self-curiosity and culture. Mexican society is an elective context to study the effects of cultural orientation on self-curiosity given the heterogeneity of its society. Although Hofstede (2001) reported Mexico as a highly collectivistic society, with a high-power distance structure, and with a relatively low tolerance for uncertainty, those characteristics do not fully apply to the entire Mexican population. Mexico embraces five regions in which marked economic and social differences exist. Not only there are significant differences in accessing economic opportunities between the North and the South, but the means of production varies significantly across Mexico between agrarian, maritime, pastoral, industrial, and digital cultures. In 10 metropolitan areas, 37.0% of the total population of the country resided in cities; the rest of the population resided in urban and rural spaces. Around 10% of the population described themselves as speaking the indigenous language or identifying themselves as having African origins. Thus, a considerable minority of the population did not primarily affiliate themselves with the westernized Mexican culture as such, with even more being multilingual ( National System of Statistical and Geographical Information, 2021).

Given the variety of Mexican society, ‘traditional’ Mexican cultural pillars such as familism/collectivism, distinctive gender roles, deference to power and authority figures and spirituality have been constantly challenged and shaped by acculturation processes with Western cultural models and by contextual demands, making “impossible to impose stereotypic characterizations to describe the behavior, attitudes, values, or beliefs of members from a particular cultural group” ( Rodriguez and Olswang, 2002, p. 157). As an example, in a sample of woman with Mexican origins, a LPA analysis highlighted “that many Mexican heritage young women disavow many of the traditional gender values that are often assumed by others to be the key components of their culture and that they do so while maintaining strong ethnic identities” ( Gutierrez and Leaper, 2022, p. 268).

Similarly, the aim of the present study was to investigate self-curiosity differences by comparing different cultural profiles found within the Mexican population, adopting both a person-centered and a variable-centered approach. More specifically, we aimed to: 1) identify cultural profiles within the Mexican sample; 2) compare, through a multigroup analysis, the structure of the SCAI-M across the cultural profiles identified; 3) investigate level differences of self-curiosity’s dimensions of Attitude and Interest in the different cultural profiles; and 4) correlate SCAI-M’s dimensions and several cultural constructs within a variable-centered approach.

Regarding the identification of cultural profiles, we expected to find similar profiles to those found in the literature. More specifically, we expected to find at least two profiles: one profile characterized by the “traditional” cultural values, similar to the “rules-based competitors” of Cooper *et al.* (2020) defined by people more collectivist, high in masculinity and avoiding uncertainty; another profile similar to the “flexible individualists” ( Cooper *et al.,* 2020), characterized by more individualistic values, and in particular by low power distance, uncertainty avoidance and collectivism and low or moderate levels of masculinity.

Regarding the cultural differences in self-curiosity, we hypothesized that individualistic people would engage in a higher self-curiosity to confirm their uniqueness and separateness from others, would describe themselves as having distinctive characteristics that are not shared by others, and would own a context-independent processing style. Conversely, profiles characterized by high masculinity could show lower scores on both self-curiosity dimensions. People not inclined to bring up their feelings when talking to others could be less likely to be open to self-exploration and interested in their inner world.

## Methods

### Ethical statement

The study was approved by the CONBIOÉTICA, the Ethical Committee of the Universidad de Monterrey (approval number 19-CEI-002-20191210). All procedures performed in studies involving human participants were in accordance with the ethical standards of the institutional research committee at the Universidad de Monterrey and with the 1964 Helsinki Declaration and its later amendments or comparable ethical standards. Written informed consent was obtained from all individual adult participants included in the study.

### Participants

528 adults participated in this study. We considered only the data of those who compiled at least the SCAI-M. Based on this criterion, we eliminated 44 subjects for a total of 484 participants (see Table 1 for sample’s characteristics). No exclusion criteria were adopted.

**Table 1.** Socio-demographic characteristics of Mexican participants (N=484).

Variable	Descriptive data	
	n	%
Gender		
Women	269	67.4
Men	130	32.6
Age range		
16-23	148	36.6
24-43	147	36.4
44-77	109	27.0
Level of education		
Compulsory education	4	1.0
High school	146	36.1
University	197	48.8
Post-graduate training	57	14.1
Employment status		
Student	163	40.3
Self-employed	64	15.8
Employee	118	29.2
Work without salary	3	0.7
Unemployed	5	1.2
Housewife/househusband	43	10.6
Retired/Pensioner	8	2.0

### Procedure

All participants voluntarily agreed to take part in the study without incentives and were recruited through mailing lists and social networks (Facebook and WhatsApp) and completed the questionnaires using the Qualtrics platform ( https://www.qualtrics.com/uk/platform/). All participants voluntarily accessed the survey and completed it online between November 9, to December 18, 2020. All data were collected in an anonymized format. No incentives were offered for participation in the study.

### Instruments

None of the scales were pilot tested prior to the start of the survey. All scales used in this study (except for SCAI-M) were structurally evaluated via confirmatory factor analysis (CFA) and explorative factor analysis (EFA). To find the best initial solutions, we performed CFA, with parallel analyses as an extraction method. The reliability of all dimensions was evaluated using McDonald’s omega (ω; Dunn *et al.,* 2014). We used Jamovi 1.6.21 version to run CFA and reliability analyses. Detailed information about psychometric analyses on the instruments are available in Appendix (see extended data, Aschieri *et al.,* 2023).

*Self-Curiosity Attitude-Interest scale, Mexican version (SCAI-M, Aschieri et al., 2021)*

Participants filled in the Mexican version of the SCAI (SCAI-M, Aschieri *et al.,* 2021). The SCAI is a seven-item scale, originally formulated by Aschieri and Durosini (2015) in Italy, composed of two factors: ASC (four items; e.g., ‘I like to listen to music because it teaches me what I am like as a person’); IKS (three items; e.g., ‘I am not interested in understanding how my past experiences impact my current life’). All items are answered on a seven-point scale, ranging from 1= completely disagree and 7 = completely agree. The psychometric properties of the SCAI-M on the present sample will be presented in the result section.

*Cultural Values Scale (CVSCALE,* *Yoo, Donthu & Lenartowicz, 2011* *)*

The instrument, composed by 26 items answered on a five-point Likert scale, measures five cultural dimensions from Hofstede model: power distance (five items; e.g., ‘People in higher positions should make most decisions without consulting people in lower positions’), uncertainty avoidance (five items; e.g., ‘It is important to have instructions spelled out in detail so that I always know what I’m expected to do’), collectivism (six items; e.g., ‘Individuals should sacrifice self-interest for the group’), long-term orientation (six items; e.g., ‘Giving up today’s fun for success in the future’), and masculinity (four items; e.g., ‘It is more important for men to have a professional career than it is for women’).

*Collective-interdependent Self-Construal Scale (SCS,* *Gabriel & Gardner, 1999* *)*

This is a 10-item scale designed to assess collective-interdependent self-construal (e.g., ‘When I am in a group, it often feels to me like that group is an important part of who I am’), which is defined as a general orientation toward representing oneself in terms of large group relationships. All items are answered on a seven-point Likert scale.

*Conformity to Masculine Norms-29 (CMN-29, Hsu & Iwamoto, 2014)*

This is a 29-item scale measuring eight factors of normative masculinity: winning (four items; e.g., ‘Winning is not my first priority’), playboy (three items; e.g., ‘If I could, I would frequently change sexual partners’), self-reliance (three items; e.g., ‘I hate asking for help’), Violence (four items; e.g., ‘I believe that violence is never justified”), heterosexual self-presentation (six items; e.g., ‘I would be furious if someone thought I was gay’), risk taking (three items; e.g., ‘I enjoy taking risks’), emotional control (three items; e.g., ‘I bring up my feelings when talking to others’), and power over women (three items; e.g., ‘Women should be subservient to men’). Items scored on a four-point Likert scale from 0 (strongly disagree) to 3 (strongly agree).

*Culture Orientation Scale (COS, Triandis and Gelfand, 1998)*

From this 16-items scale designed to measured four dimensions of collectivism and individualism we selected only the dimension of horizontal collectivism (four items; e.g. ‘The well-being of my coworkers is important to me’) because of poor fit of the whole factorial structure of the scale. All items were answered on a nine-point Likert scale.

*Relational-Interdependent Self-Construal Scale (Rel-IntSC,* *Cross, Bacon, and Morris, 2000* *)*

This is an 11-item scale designed to assess relational-interdependent self-construal, defined as a general orientation toward representing oneself in terms of close relationships (e.g., ‘My close relationships are an important reflection of who I am’). In the present study, only nine items were retained to obtain acceptable fit of the scale structure. Items 8 and 9 (‘Overall, my close relationships have very little to do with how I feel about myself’, and ‘My close relationships are unimportant to my sense of what kind of person I am’) were excluded to allow good fit of the scale to the data (see Appendix). All items were answered on a seven-point Likert scale.

*Sixfold Self-Construal Scale (SSC,* *Harb and Smith, 2008* *)*

This is a 30-item scale measuring six subcategories of self-construal: the personal self (five items; e.g., ‘I am a unique person separate from others’); relational vertical (five items; e.g., ‘I think of myself as connected to my family’); relational horizontal (five items; e.g., ‘I control my behavior to accommodate the wishes of my friends’); collective vertical (five items; e.g., ‘I am affected by events that concern to my social organization’); collective horizontal (five items; e.g., ‘I am aware of the needs, desires, and goals of my peers), and humanity (five items; e.g., ‘I feel I have a strong relationship with humankind’). The subdimension of personal self was dropped due to psychometric problems. All items were answered on seven-point Likert-type scales.

### Data collection and analysis

The missingness mechanism was evaluated by Little’s MCAR test on the entire set of measures administered, indicating that data were not distributed randomly [ *χ* ^2^ (1541)=1699.40; p=.003]. Missing data were handled in Mplus 8.4 software ( https://www.statmodel.com/) via the full information maximum likelihood (FIML) method.

To identify different profiles of cultural orientation among participants based on Hofstede’s five-dimension model, we conducted a latent profile analysis (LPA; Collins and Lanza, 2013) using Mplus 8.4. The core assumption of LPA is that individuals are homogeneous within the same profile and dissimilar between the different profiles, with respect to the mean level of the considered indicators. LPA was performed on the five dimensions of the cultural values scale *(CVS),* for a total of 15 observed indicators, comparing increasing number of profiles (k). The decision about the best profile solution was based on fit indices, parsimony, classification precision and interpretability of the profiles ( Marsh *et al.,* 2009).

Then, we tested a CFA of the SCAI-M on the total sample using MLR as the extraction method. To test the measurement invariance of the SCAI-M in relation to the profiles of cultural orientation, a series of multigroup CFA were tested in a step-by-step process. First, we tested the configural invariance of the two-factor structure of SCAI-M. Second, we tested metric invariance by constraining the factor loadings to be equal across the different profiles of cultural orientation. The metric invariance model was then compared with the configural one. Full metric invariance is found when a chi-square difference is non-significant (changes were calculated using the Satorra-Bentler Scaled; www.statmodel.com/chidiff.shtml), the CFI’s decrease is lower than .01, and the RMSEA’s increase is equal to or lower than .015 ( Chen, 2007). Following the same procedure, we tested a scalar invariance model in which equality constraints were added on intercepts across the different profiles; this model was compared with the metric invariance model using the same cut-off scores. When full measurement invariance was not found, modification indexes were consulted to remove parameter constraints until reaching satisfactory cut-offs, to obtain a model of partial invariance. We used Mplus 8.4 version to run multi-group confirmatory factor analyses.

Finally, adopting a variable-centered approach, we also evaluated whether the two dimensions of the SCAI-M were associated to several cultural variables. The ‘diffcor’ R package was used for correlations and between-groups comparisons.

All analyses can be run with non-proprietary software. Here follows a freely accessible alternative software capable of the same analyses. LPA models can be fit in *R* (version 4.0.3; R-core team, 2020), running in *Rstudio* (version 1.3.1093), and the package ‘ *tidyLPA*’. CFA and multigroup CFA can be run using *R* (version 4.0.3; R-core team, 2020), running in *Rstudio* (version 1.3.1093), and the *lavaan* package.

## Results

Analyses were carried on a total of 484 respondents (female, n = 269), with age ranging from 16 to 23 years old (36.6%), 24 to 43 years old (36.4%) and 44 to 77 (27%). Most of participants were students (40.3%) and employees (29.2%) ( Aschieri *et al.,* 2023). Overall our sample was educated, with 62.9% participants having a university degree of higher title of study, and only 1% having finished compulsory education.

### Latent profile analyses

**Figure 1.**
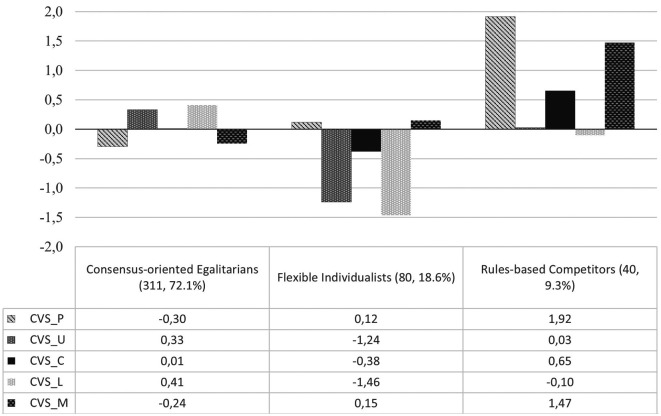
Representation of the three profiles of cultural orientation based on Hofstede’s model expressed as mean deviations on CVS five indicators. *Note.* CVS=Cultural Values Scale (P=power distance, U=uncertainty avoidance, C=collectivism, L=long term orientation, M=masculinity.

We compared three measurement models, and we stopped in correspondence with the four-profile solution as this model was not identified (Table S1 in Supplementary materials; Aschieri *et al.,* 2023). The three-profile model turned out to be the preferred one according to all the criteria (AIC, BIC, CAIC, AWE; BLRT; Entropy; Profiles’ distribution), and to the inspection of the classification-diagnostics criteria (Table S2 in Supplementary materials; Aschieri *et al.,* 2023). Figure 1 presents the profiles’ solution with standardized values on the y-axis, indicating each profile’s mean deviation from the total sample mean. Specifically, the largest group was made of individuals who scored on average on all the items administered (i.e., their scores were included in the range of 0.5 standard deviations from the sample mean), thus they were named ‘consensus-oriented egalitarians’, as they were like the people found by Cooper *et al.* (2020) as characterized by high collectivism and uncertainty avoidance and low power distance and masculinity. The second group was made of individuals with very low uncertainty avoidance and low long-term orientation, thus they were named ‘flexible individualists’, as they were similar to people belonging to the profile found by Cooper *et al.* (2020) which was characterized by low power distance, uncertainty avoidance and collectivism and moderate masculinity *.* Conversely, the last group represented individuals with extreme scores for power distance and masculinity, and high scores for collectivism. This last group was named ‘rules-based competitors’, as it was like the one found by Cooper *et al.* (2020) characterized by high power distance, masculinity, uncertainty avoidance and collectivism *.*

### SCAI-M measurement model and invariance across cultural profiles

On the total sample, the SCAI-M showed acceptable fit with the expected two-factors structure (χ ^2^/df=3.5; CFI=.917; RMSEA=.071 [C.I. .051 - .093] and acceptable reliability coefficients for ASC (four items, ω=.69) and IKS (three items, ω=.69). When multigroup analyses were conducted, the configural invariance model showed acceptable fit indexes (χ ^2^/df=2.25; CFI=.890; RMSEA=.093), so the two-factors structure could be considered suitable for the three cultural profiles. However, when metric invariance was tested, the model resulted in a significant decrease of the fit (Δχ ^2^ (13)=54.82, p<.001; ΔCFI=-.103; ΔRMSEA=.020). An inspection of modification indexes revealed that factor loadings of items four and seven were not similar in the three groups. A partial metric invariance model was then tested by removing the equality constraints on the item four factor loading. Moreover, the factor loading of item seven in the consensus-oriented egalitarian group was set free to account for its different weight in contributing to the IKS (Δχ ^2^ (11)=14.19, p=.223; ΔCFI.000; ΔRMSEA=-.010). Items’ factor loading estimates revealed that the consensus-oriented egalitarian group presented a lower factor loading of item four (‘I select my best friends among those with whom I can grow as a person’; factor loading=0.407) than rules-based competitors (.809), and flexible individualists (1.411). Thus, the meaning of the attitude dimension was more related to social relationships for the rules-based competitors and, especially, for flexible individualists, whereas, for consensus-oriented egalitarians, attitude was more related to individual preferences and less to relational choices. Regarding item seven (‘I am not interested in understanding what motivates my behaviors.’), the consensus-oriented egalitarians group presented a lower factor loading (.916) than the other two groups (1.829), showing that, also in this case, the (absence of) interest dimension was more defined by item seven for the flexible individualists and rules-based competitors groups than for the consensus-oriented egalitarians ^1^.

Finally, a scalar invariance model was tested showing a significant decrease of the fit (Δχ ^2^ (9)=25.24, p=.003; ΔCFI=-.033; ΔRMSEA=.004). An inspection of modification indexes revealed that the intercepts of item four were not similar between the consensus-oriented egalitarians group and the other two groups, so a partial scalar invariance model was tested releasing the intercept of item four only for the consensus-oriented egalitarians group (Δχ ^2^ (8)=11.42, p=.179; ΔCFI=-.010; ΔRMSEA=-.003). Estimates revealed that consensus-oriented egalitarians presented a higher intercept of item four (6.165) than the other two groups (5.643), indicating that scores on that item depended on the different way respondents used the 1-7 scale for item four. That means that those people were more interested in selecting their friends among people who allow them to grow than people belonging to flexible individualists or rules-based competitors profiles.

### Correlations between SCAI and cultural dimensions

Table 2 shows the correlations between SCAI-M total scale, SCAI-M Attitude and SCAI-M Interest factors and the cultural dimensions measured by the other scales considered in this study. SCAI-M total scale strongest positive correlations were with relational-interdependent self-construal and horizontal collectivism. Smaller correlations were found with collective-interdependent self-construal, humanity, relational horizontal, relational vertical, and, in a negative direction, with emotional control. SCAI-M Attitude strongest positive correlation was with relational-interdependent self-construal, whereas smaller correlations were with horizontal collectivism, collective-interdependent self-construal, relational horizontal, collective vertical, collective horizontal, and humanity. SCAI-M Interest strongest positive correlation was with Relational interdependent self-construal, whereas smaller positive correlation with Horizontal collectivism, and negative correlations with heterosexual self-presentation, emotional control, and power over woman were found.

**Table 2.** Summary table of Pearson’s correlation coefficients.

Scale’s factors	M (SD)	SCAI Attitude	SCAI Interest	SCAI Total scale	*p*-value (Fisher Z-test) ^a^
SCAI attitude	5.10 (1.21)	1	.221 ***		
SCAI interest	5.72 (1.36)	.221 ***	1		
COS					
Horizontal collectivism	8.09 (1.21)	.308 ***	.307 ***	.402 ***	1.00 (N=475)
REL-IntSC					
Relational-interdependent self-construal	5.34 (.95)	.435 ***	.358 ***	.522 ***	.133 (N=455)
SCS					
Collective-interdependent self-construal	4.80 (1.10)	.324 ***	.142 **	.312 ***	.001 (N=459)
SSC					
Relational vertical	5.80 (1.08)	.166 ***	.157 ***	.216 ***	.876 (N=451)
Relational horizontal	5.25 (1.16)	.304 ***	.151 ***	.305 ***	.007 (N=451)
Collective vertical	4.34 (1.61)	.210 ***	.069	.194 ***	.015 (N=451)
Collective horizontal	4.50 (1.48)	.232 ***	.044	.192 ***	.001 (N=451)
Humanity	5.12 (1.24)	.301 ***	.107 *	.276 ***	.001 (N=451)
CMN-29					
Winning	2.37 (.74)	-.102 *	-.020	-.085	.167 (N=444)
Playboy	1.58 (.80)	-.018	-.162 ***	-.111 *	.015 (N=444)
Self-reliance	2.26 (.76)	.020	-.117 *	-.053	.101 (N=444)
Violence	1.78 (.74)	-.180 ***	-.100 *	-.188 ***	.172 (N=444)
Heterosexual self-presentation	2.27 (.77)	-.111 *	-.210 ***	-.196 ***	.090 (N=444)
Risk taking	2.48 (.68)	.135 **	.016	.106 *	.044 (N=444)
Emotional control	2.32 (.84)	-.207 ***	-.286 ***	-.309 ***	.164 (N=444)
Power over woman	1.47 (.66)	-.067	-.288 ***	-.211 ***	<.001 (N=444)

* p<.05.

** p<.01.

*** p=.001.

^a^ Fisher's z-tests were computed with the ‘diffcor’ R package ( https://CRAN.R-project.org/package=diffcor) to test differences between correlations of scale’s factors with SCAI attitude and SCAI interest.

## Discussion

The principal focus of this study was to investigate cultural differences in self-curiosity measurement by adopting both a person-centered and variable-centered approach. Regarding the person-centered approach, three cultural profiles within the Mexican sample were identified through LPA: consensus-oriented egalitarians were people with average scores on all cultural dimensions, flexible individualists were people that appreciated uncertainty and short-term orientation, and rules-based competitors people showed very high scores in power distance, masculinity, and collectivism. As expected, the profiles were similar to the ones identified by Cooper *et al.* (2020), although the prevalence of the profiles within the Mexican sample was different especially for the consensus-oriented egalitarians’ profile, that was more represented in our sample than in Cooper *et al.* (2020), and the rules-based competitors, that was less represented in our sample. Those findings support the variety of cultural profiles present in Mexico and confirm the importance to evaluate within-country cultural differences ( Taras *et al.,* 2016). Moreover, Cooper *et al.* (2020) did not use the dimension of Long/Short Term Orientation to identify latent cultural profiles, so the present study is the first that considers that dimension to enrich the description of the cultural profiles within one country.

Adopting the person-centered approach highlighted some cultural differences in the measurement of self-curiosity that were not previously found. Indeed, beside partial scalar invariance found also in previous cross-country comparisons ( Durosini *et al.,* 2018; Aschieri *et al.,* 2021), also partial metric invariance was found. This suggests that the meaning of ASC is more related to the choice of friends for the flexible individualists and rules-based competitors than for the consensus-oriented egalitarians. Moreover, flexible individualists and rules-based competitors perceive IKS more in terms of motivational features than do the consensus-oriented egalitarians. Some differences were also present regarding the mean level of item four related to social relationships, with consensus-oriented egalitarians that scored higher than the other two groups.

SCAI-M is more likely to provide stable assessment results of self-curiosity traits when respondents do not position on the tails of the distribution of cultural dimensions. When this happens, the results should be interpreted with caution, since the relevance that respondents give to their friends in exploring their inner world assumes a unique and higher relevance in the measurement of their self-curiosity. Thus, professionals aiming to promote self-curiosity in traditionalist clients should invest efforts to build relationships with them and join their traditionalist point of view as the first step of their work. This seems coherent with Eriksen *et al.,* (2002), who described the difficulties with conservative Christian clients, and suggested building a solid relationship first, by empathically placing themselves in the client’s shoes, and being open to learn about their worldview. Thus, adopting a person-centered approach in evaluating the cultural profiles helped to understand that cross-cultural differences in the measurement of the construct of self-curiosity need to be considered even when comparisons are made within one country.

Adopting the variable-centered approach showed that the SCAI-M was negatively correlated with power-related definitions of masculinity, and, in particular, to its emotional control component. Contrary to the expectations, the SCAI-M positively correlated with collectivism, with higher correlation coefficients with relational interdependence in the definition of the respondents’ identity. The SCAI-M was associated with the respondents’ ability to conceptualize their identity as strictly connected to a relational network. It might be speculated that self-curiosity can help respondents to make sense of the continuity of their identity in the context of the different people they relate with, and to be more aware of the interconnectedness between their current self-identity and their relational network. However, those findings could also be affected by some issues related to the measurement of cultural variables, indeed, as seen in the Appendix ( Aschieri *et al.,* 2023), some of the scales had less than acceptable model fit in the CFA.

### Limits and future perspectives

This study tested the structure of the SCAI-M in three groups of Mexican respondents. While the consensus-oriented egalitarians group was large and findings on the SCAI-M metrics with these respondents were reliable, the other two groups were smaller. Thus, to confirm the findings on flexible individualists and rules-based competitors, future research focusing on these marginal cultural orientations are needed.

Regarding the correlation between the SCAI-M’s dimensions and other cultural variables, we should be cautious in the interpretation because of the unsatisfactory fit of many of the instruments used, moreover, we cannot rule out the possible correlations with other cultural dimensions not considered in this study. Furthermore, future additional studies are necessary to increase the generalizability of LPA results obtained with groups collected through convenience sampling, and resulting featured by high levels of education.

Testing the measurement properties of the SCAI with a clinical population is also compelling. Methodologically, LPA could be used to describe the profiles of specific groups of patients including both measures of clinical symptoms and self-curiosity. Results of this typology of studies would allow tailoring interventions to specific typologies of patients by increasing the understanding of their interest and attitude to understand themselves better.

Overall, the present study contributes to both the self-curiosity and cross-cultural literature in several ways. It confirmed that within a country it is possible to find cultural variability, but also proposes an approach to deal with such variability: the use of both person-centered and variable-centered approach in investigating cultural differences. Self-curiosity measurement was also confirmed to be linked with the cultural features, adding that when culture is investigated more in deep than only comparing countries, new findings could rise. Thus, future studies aiming at examining cross-cultural differences in the use of measurement tools could benefit of that approach.

## Data Availability

Repository name: Cultural differences in the measurement of self-curiosity within Mexico: a person-centered and variable-centered study. https://doi.org/10.17632/zvcc9gvys9.1 ( Aschieri *et al., *2023). The project contains the following underlying data:
• Dataset.sav (List of all variables analyzed). • Codebook (Items/constructs corresponding to each variable in the dataset). Repository name: Cultural differences in the measurement of self-curiosity within Mexico: a person-centered and variable-centered study. https://doi.org/10.17632/zvcc9gvys9.1 ( Aschieri *et al., *2023). This project contains the following extended data:
• Supplementary (Appendix: Psychometric properties of all instruments used in the study; Syntax of the analyses; Supplementary Tables 1 and 2) • Questionnaire/Cuadernillo (list of the items corresponding to columns the dataset) Repository: SRQR checklist for ‘Cultural differences in the measurement of self-curiosity within Mexico: a person-centered and variable-centered study’. https://doi.org/10.17632/zvcc9gvys9.1 ( Aschieri *et al., *2023). Data are available under the terms of the Creative Commons Attribution 4.0 International license (CC-BY 4.0).

## Footnotes

^1^
The residual of item seven had to be constrained to 0 for the rules-based competitors group, because otherwise, it generated a negative value.
