# Supplementary material for: Cultural differences in the measurement of self-curiosity within Mexico: a person-centered and variable-centered study
Source: F1000Res. 2023 Sep 18;12:1168. [Version 1] doi: 10.12688/f1000research.140151.1 (PMC10638485; doi:10.12688/f1000research.140151.1)
Supplement: Peer review history

# Reviewer report: Reviewer response for version 1

**DOI:** 10.5256/f1000research.153481.r217960

**Published:** 2023-11-10

**Authors:** Stefania Cristofanelli, Eleonora Centonze

**Affiliations:** 1 Department of Social and Human Science, Università della Val D'Aosta, AOSTA, Italy

The aim of the research is to evaluate whether, and possibly how, the culture influences the attitude of curiosity and interest in increasing self-knowledge. To achieve this goal, the authors adopted both a person-centered and a variable-centered approach. The personal disposition to be curious was investigated using the Self-Curiosity Attitude-Interest scale Mexican version (SCAI-M); moreover, five other questionnaires were administered to evaluate further cultural dimensions. The sample was composed of 484 Mexican adults; the data collected were analyzed using a multigroup confirmatory factor analysis and a latent profile analysis. The choice to select a Mexican sample has the advantage of analyzing a social context within which different cultures coexist. The latent profile analysis revealed the presence of three different cultural profiles: flexible individualists, rule-based competitors and consensus-oriented egalitarians. At a clinical level, this result suggests that it is important, even within the same country, to consider the culture of the individual when exploring the construct of self-curiosity. By adopting the person-centered approach, it emerged that in flexible individualists and rule-based competitors profiles, the attitude of self-curiosity was associated with the choice of friends. From a clinical point of view, this data is interesting, because it suggests that when a therapist should promote an attitude of curiosity in a traditionalist client the professional should begin by building a relationship based on adopting the patient's point of view. Furthermore, in flexible individualists and rule-based competitors, the interest in self-knowledge is more understood in terms of motivational characteristics. Adopting the variable-centered approach, the SCAI-M scale negatively correlates with the definitions of masculinity most related to power and, in particular, to its emotional control component. Contrary to expectations, SCAI-M correlates positively with collectivism.

 The paper is interesting because it places emphasis on how culture influences the attitude of curiosity towards oneself, an internal disposition which, in the clinical field, is crucial both for understanding the functioning of the individual and for change (Kamphuis, & Finn, 2018). Furthermore, the study warns against the mistake of not considering the impact of different cultures even among individuals living within the same country (Taras et al., 2016).

 Suggestions:

- In the introduction section can be add a brief description of the person-centered approach and the variable-centered approach.
- As stated in the footnote (page 8), in one case the residual had to be constrained to zero because otherwise it generates a negative value. From the literature you consulted, are there any other studies in a similar condition?
- Could the SRMR (Standardized Root Mean Square Residual) also be reported in the CFA results as a measure of fit?

Is the work clearly and accurately presented and does it cite the current literature?

Yes

If applicable, is the statistical analysis and its interpretation appropriate?

Yes

Are all the source data underlying the results available to ensure full reproducibility?

Yes

Is the study design appropriate and is the work technically sound?

Yes

Are the conclusions drawn adequately supported by the results?

Yes

Are sufficient details of methods and analysis provided to allow replication by others?

Yes

Reviewer Expertise:

a) Research concerns diagnostic assessment, with a particular interest in the clinical-diagnostic aspects of psychopathology in adolescence, b) the evaluation of outcomes and processes in the care pathways of adolescent patients placed in therapeutic and socio-rehabilitative communities.

We confirm that we have read this submission and believe that we have an appropriate level of expertise to confirm that it is of an acceptable scientific standard.

## References

[1] : Therapeutic Assessment in Personality Disorders: Toward the Restoration of Epistemic Trust. *Journal of Personality Assessment* .2019;101(6) : 10.1080/00223891.2018.1476360 662-674 10.1080/00223891.2018.1476360 29873526

[2] : Does Country Equate with Culture? Beyond Geography in the Search for Cultural Boundaries. *Management International Review* .2016;56(4) : 10.1007/s11575-016-0283-x 455-487 10.1007/s11575-016-0283-x

# Reviewer report: Reviewer response for version 1

**DOI:** 10.5256/f1000research.153481.r207990

**Published:** 2023-10-09

**Authors:** Juan Manuel Fernández-Cárdenas

**Affiliations:** 1 Tecnologico de Monterrey, Monterrey, Nuevo Leon, Mexico

The article aims to investigate the relationship of self curiosity with possible cultural traits in Mexican population. The study found correlation of self-curiosity with three previously found cultural profiles. These profiles are found in different degrees within the Mexican sample studied, confirming that a national culture cannot be assumed as relatively homogenous. The study is relevant for clinical implications, as self-curiosity is a very valuable trait for psychotherapy, given that this allows patients to think and feel about his or her mental states in an exploratory mode, allowing the possibility of alternative explanations about the behavior of others towards him or her, and the resulting feelings he or she can develop as a consequence of those behaviors. See the work of Peter Fonagy and colleagues in terms of mentalization (e.g. 2003), where curiosity about how the mind of others and your own mind works, is a key element for the development of a reflective function, and a key ingredient for mental health. If curiosity can be conceptualized also as a cultural trait, then a psychotherapist could be more aware about the need for constructing more carefully relationships with patients who might need more trust to be developed (Fonagy and Campbell, 2023) in order to feel more secure about disclosing possible hypotheses of mental states that could be felt as more traditional or rigid, and therefore with possible links to shame or guilt, for instance. This is a very interesting article, very useful to identify tests that can reliably measure these traits, and thus promote more effective ways for clinical practice.

Is the work clearly and accurately presented and does it cite the current literature?

Yes

If applicable, is the statistical analysis and its interpretation appropriate?

I cannot comment. A qualified statistician is required.

Are all the source data underlying the results available to ensure full reproducibility?

Yes

Is the study design appropriate and is the work technically sound?

Yes

Are the conclusions drawn adequately supported by the results?

Yes

Are sufficient details of methods and analysis provided to allow replication by others?

Yes

Reviewer Expertise:

Sociocultural research in psychology and education, linguistics and ethnography, dialogue and clinical practice

I confirm that I have read this submission and believe that I have an appropriate level of expertise to confirm that it is of an acceptable scientific standard.

## References

[1] : Affect regulation, mentalization and the development of the self. *Karnac Books* .2003;

[2] : Epistemic trust and mistrust in helping systems. In Adaptive Mentalization-Based Integrative Treatment (AMBIT) for People with Multiple Needs: Applications in Practice. *Oxford University Press* .2023;

# Response

**Published:** 2023-11-05

**Authors:** Semira Tagliabue

**Affiliations:** Universita Cattolica del Sacro Cuore, Brescia, Lombardy, Italy

Thank you for the warm and supportive feedback on our work.

 We are encouraged to move ahead in this field by your kind words of appreciation.

 Semira Tagliabue, Filippo Aschieri and co-authors
